# Efficient Photocatalytic Bilirubin Removal over the Biocompatible Core/Shell P25/g-C_3_N_4_ Heterojunctions with Metal-free Exposed Surfaces under Moderate Green Light Irradiation

**DOI:** 10.1038/srep44338

**Published:** 2017-03-13

**Authors:** Shifei Kang, Hengfei Qin, Lu Zhang, Yongkui Huang, Xia Bai, Xi Li, Di Sun, Yangang Wang, Lifeng Cui

**Affiliations:** 1 Department of Environmental Science and Engineering, University of Shanghai for Science and Technology, Shanghai, 200093, China; 2 School of Chemical and Environmental Engineering, Jiangsu University of Technology, Changzhou, 213001, China; 3 Department of Environmental Science and Engineering, Fudan University, Shanghai 200433, China; 4 Department of Ultrasound in Medicine, Shanghai Jiao tong University Affiliated Sixth People’s Hospital, Shanghai Institute of Ultrasound in Medicine, Shanghai 200233, China

## Abstract

Highly-monodispersed g-C_3_N_4_/TiO_2_ hybrids with a core/shell structure were synthesized from a simple room temperature impregnation method, in which g-C_3_N_4_ was coated through self-assembly on the commercially available Degussa P25 TiO_2_ nanoparticles. Structural and surface characterizations showed that the presence of g-C_3_N_4_ notably affected the light absorption characteristics of TiO_2_. The g-C_3_N_4_/TiO_2_ heterojunctions with metal-free exposed surfaces were directly used as biocompatible photocatalysts for simulated jaundice phototherapy under low-power green-light irradiation. The photocatalytic activity and stability of g-C_3_N_4_/TiO_2_ were enhanced relative to pure P25 or g-C_3_N_4_, which could be ascribed to the effective Z-scheme separation of photo-induced charge carriers in g-C_3_N_4_/TiO_2_ heterojunction. The photoactivity was maximized in the 4 wt.% g-C_3_N_4_-coated P25, as the bilirubin removal rate under green light irradiation was more than 5-fold higher than that under the clinically-used blue light without any photocatalyst. This study approves the future applications of the photocatalyst-assisted bilirubin removal in jaundice treatment under moderate green light which is more tolerable by humans.

Neonatal jaundice, which is caused by excessive bilirubin retention in skin and blood, has become one major threat to the health of newborn babies^1^,^2^. So far, many therapeutic protocols have been developed to overcome its damages to the central nervous system and among them, the commonly-used protocol for neonatal hyperbilirubinemia is the phototherapy under blue light (430–490 nm) or green light (490–570 nm) irradiation^3^. The ideal irradiation therapy is expected to degrade a large part of blood bilirubin and thereby relieve the harms to nearby healthy tissues. In clinical phototherapy, the blue light with high energy is widely used as an irradiation source to achieve satisfactory degradation. However, hemoglobin, which is abundant in the human body, can also strongly absorb blue light irradiation and thus compete with bilirubin for this light, thereby reducing the phototherapeutic efficacy. This means a blue light therapy apparatus only with high optical intensity is feasible. Therefore, American Academy of Pediatrics recommends high-intensity phototherapy under blue light (430–490 nm) for neonatal hyperbilirubinemia. Moreover, blue light sources are always incorporated with ultraviolet light, which is significantly harmful to human eyes and skin and causes DNA damages. With provision that most receivers of phototherapy are the jaundiced newborn infants, the irradiation time should be strictly controlled, thus making the phototherapy time-consuming and agonizing. Even the blue light source of light- emitting diode (LED), which is regarded as a low-intensity light source, cannot eliminate the side effects such as hyperthermia, insensible water loss and rash^4^.

With the popularization of phototherapy, its clinical value and safety have attracted growing attention. Comparatively, the green light irradiation is more moderate and biotolerable, since the only question is how to improve the phototherapy efficiency under this green light irradiation. In this regard, biocompatible visible-light-driven (VLD) photocatalysts that are specially designed for green-light bilirubin phototherapy are very attractive^5^,^6^,^7^. However, most of VLD photocatalysts are metal oxide or metal sulfide semiconductors. Even some metal species such as Au and iron oxide have been used in therapeutics research, their clinical applications *in vivo* are still limited by the potential risks of dissolved toxic ions and immune response induced by exogenous photocatalysts. Therefore, attention has been paid to the efficient and stable VLD photocatalysts with metal-free exposed surfaces because of their improved biosafety^8^,^9^. Recently, graphitic carbon nitride (g-C_3_N_4_), a typical layered-stacked metal-free polymer semiconductor with a narrow band gap of 2.7 eV, has attracted intensive attention for its promising applications as a VLD photocatalyst owing to its advantages such as low cost, nontoxicity and stability within PH 0–14^10^,^11^. Unfortunately, the overall photocatalytic efficiency of pure g-C_3_N_4_ is limited by its low nonlocalized conductivity, high recombination rate and small specific surface area^12^,^13^. Interestingly, the photocatalytic activity of g-C_3_N_4_ can be significantly improved by incorporating g-C_3_N_4_ with other semiconductors (e.g. g-C_3_N_4_/TiO_2_ and g-C_3_N_4_/AgCl) to form photocatalytic heterojunctions^14^,^15^,^16^,^17^. Thus, g-C_3_N_4_ inspired low-toxicity semiconductor heterojunctions are a good choice for phototherapy of jaundice. Considering future applications *in vivo*, we think the surfaces of the designed photocatalyst for bilirubin phototherapy must be covered by metal-free g-C_3_N_4_ through close-knit. Thus, we chose g-C_3_N_4_-coated TiO_2_ as the ideal phototherapy photocatalyst.

Herein, we demonstrate a facile self-assembly impregnation route for synthesis of g-C_3_N_4_/TiO_2_ heterojunctions with different g-C_3_N_4_ contents, and employ them for photocatalytic bilirubin removal for the first time. The metal-free g-C_3_N_4_ coating layer on the surface of P25 TiO_2_ plays an important role in the enhanced photocatalytic performance, and shows great potential in future application of phototherapy for neonatal hyperbilirubinemia. The photocatalytic performances of g-C_3_N_4_/TiO_2_ photocatalysts were evaluated for bilirubin degradation using a narrow-band green LED light source. Meanwhile, the application potential of photocatalyst-assisted green light phototherapy was discussed and compared with the conventional high-intensity blue light phototherapy.

## Results and Discussion

### Schematic illustration of the synthesis process

**Figure 1:**
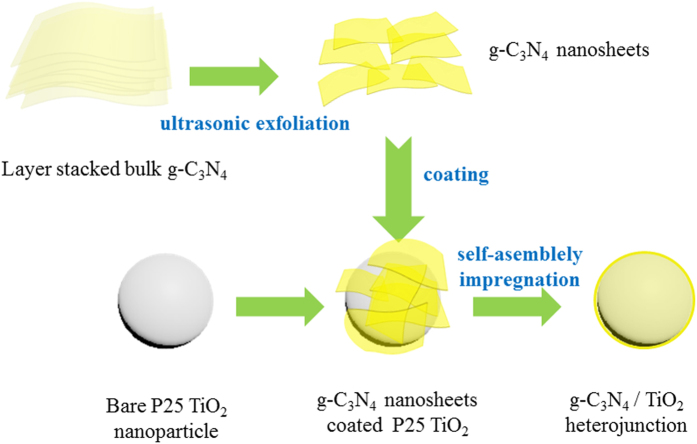
Schematic illustration of the synthesis of g-C_3_N_4_/TiO_2_ heterojunctions by a self-assembly impregnation route.

Figure 1 shows this simple self-assembly impregnation route for synthesis of g-C_3_N_4_/TiO_2_ heterojunctions. During the process, the commercially available P25 TiO_2_ nanoparticles was considered as a host and template for the self-assembly of g-C_3_N_4_ nanosheets, while the cheap and recyclable melamine was used as the sole dispersion liquid. As reported, in methanol, stack- layered g-C_3_N_4_ can easily be exfoliated into thin nanosheets, which are prone to a rolling and regrowth process^14^. In this impregnation, the g-C_3_N_4_ nanosheets could curl up and wrap around the P25 TiO_2_ nanoparticles to minimize the total interfacial energy, and then reassemble into a homogeneous coating layer after the methanol was removed by the air stream. The main advantage of this method is that the entire composite process is waste-free and achievable at room temperature. Nevertheless, introducing a very low amount of g-C_3_N_4_ (<10 wt.%) can stabilize the metal-free surfaces of photocatalysts. Because of environmental friendliness, low cost and high yield, this method is appealing for future application of photocatalyst-assisted phototherapy of neonatal hyperbilirubinemia. The g-C_3_N_4_/TiO_2_ heterojunctions as-prepared were named as PCNx, where *x* is the weight percentage of g-C_3_N_4_.

### Crystal structure

**Figure 2:**
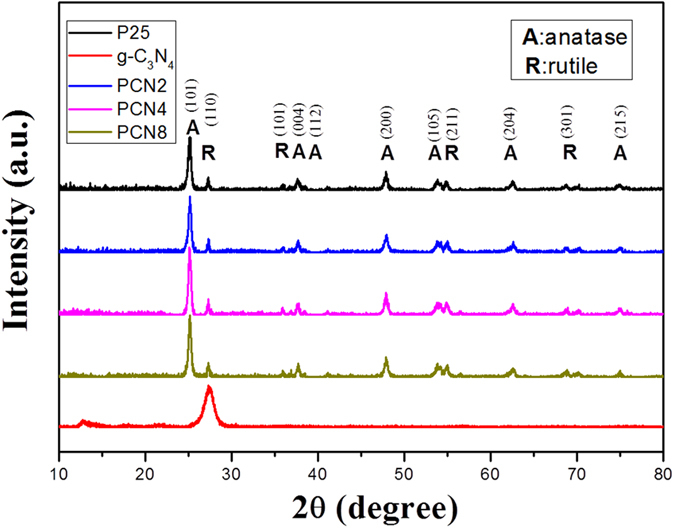
XRD patterns of P25 TiO_2_, pure g-C_3_N_4_, PCN2, PCN4 and PCN8.

Figure 2 shows the X-ray diffraction (XRD) patterns of PCNx. The diffraction peaks at 2*θ* = 25.3, 37.7, 47.9, 53.8, 62.6 and 74.9° can be ascribed to (1 0 1), (0 0 4), (2 0 0), (1 05), (2 0 4) and (2 1 2) reflection of anatase TiO_2_, respectively, while the peaks at 2*θ* = 27.8, 35.9, 54.9 and 68.8° can be ascribed to (1 1 0), (1 0 1), (2 1 1) and (3 0 1) reflection of rutile TiO_2_, respectively^18^,^19^. The patterns clearly illustrate that the crystals of PCN2, PCN4 and PCN8 are all composed of 80% anatase TiO_2_ and 20% rutile TiO_2_, which are nearly the same as P25 TiO_2_. The predominant anatase phase in P25 TiO_2_ was sustained after g-C_3_N_4_ modification. The unique mixcrystal structure of P25 TiO_2_ is considered to be favorable for photocatalysis applications. Since the anatase-to-rutile phase transformation of TiO_2_ occurs at around 600 °C, the low-temperature treatment benefits the crystal stability of P25 TiO_2_-based photocatalyst. Pure g-C_3_N_4_ shows two distinct diffraction peaks at 2*θ* = 13.2° and 27.4°, corresponding to the (1 0 0) and (0 0 2) peaks of the graphitic phase, respectively^20^. However, the XRD patterns of PCNx are not changed notably after coating with g-C_3_N_4_ because of the low content (<8%) and the low XRD intensity of g-C_3_N_4_. Nevertheless, the co-presence of TiO_2_ and g-C_3_N_4_ was confirmed by X-ray photoelectron spectroscopy (XPS) (Figure S1). Besides, PCNx have the similar average crystal sizes as P25, demonstrating the encapsulation of g-C_3_N_4_ layers also suppresses the growth of TiO_2_ crystals under the impregnation coating treatment.

### Nitrogen adsorption analysis

**Figure 3:**
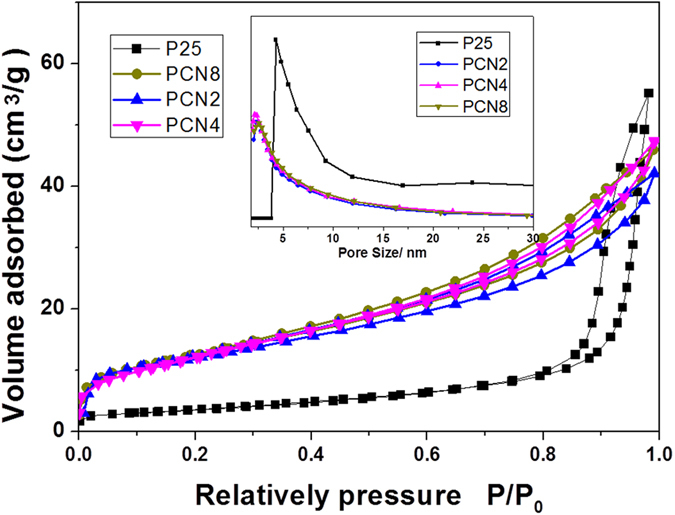
Nitrogen adsorption–desorption isotherms of P25 TiO_2_, PCN2, PCN4 and PCN8. Inset shows the corresponding pore size distributions of P25 TiO_2_ and PCN4 calculated by the BJH method.

The specific surface areas (SSAs) of PCNx were determined from Nitrogen adsorption and desorption isotherms (Fig. 3). The insets in Fig. 3 show the Barrett-Joyner-Halenda (BJH) pore distributions of pure P25 TiO_2_ and PCNx. Unlike pure P25 TiO2, the isotherm curves of the PCN2, PCN4 and PCN8 exhibit a distinct uptake of N2 as a result of capillary condensation in a wide relative pressure (P/P0) range of 0.4–0.95, which indicates the existence of multiform pore distributions. The low-pressure hysteresis loop (0.4 < P/P_0_ < 0.8) is related to the intra-aggregated pores of g-C_3_N_4_–TiO_2_, while the high-pressure hysteresis loop (0.8 < P/P_0_ < 0.95) is probably associated with the larger pores formed between secondary particles^21^. The Brunauer–Emmett–Teller SSAs (S_BET_), pore volumes, and pore sizes of all samples are summarized in Table 1. The S_BET_ of PCNx as well as the pore volumes decreases slightly compared to pure P25 TiO_2_, which is ascribed to the adhesion and self-aggregation of P25 nanoparticles in liquids during impregnation. Nevertheless, the S_BET_ of the representative PCN4 is up to 45.79 m^2^·g^−1^, which is favorable compared with other reports of TiO_2_ based TiO_2_/g-C_3_N_4_ photocatalysts^22^,^23^. The S_BET_ of show slightly variations with g-C_3_N_4_ mass ratio increased, in which PCN8 sample with a highest g-C_3_N_4_ content processed a lower S_BET_ compared with that of PCN4, the possible reason was that the excessive g-C_3_N_4_ nanosheets cannot effectively hybrids with P25 nanoparticles and agglomerate into g-C_3_N_4_ cluster with low specific surface areas.

### Morphologic characterization

**Figure 4:**
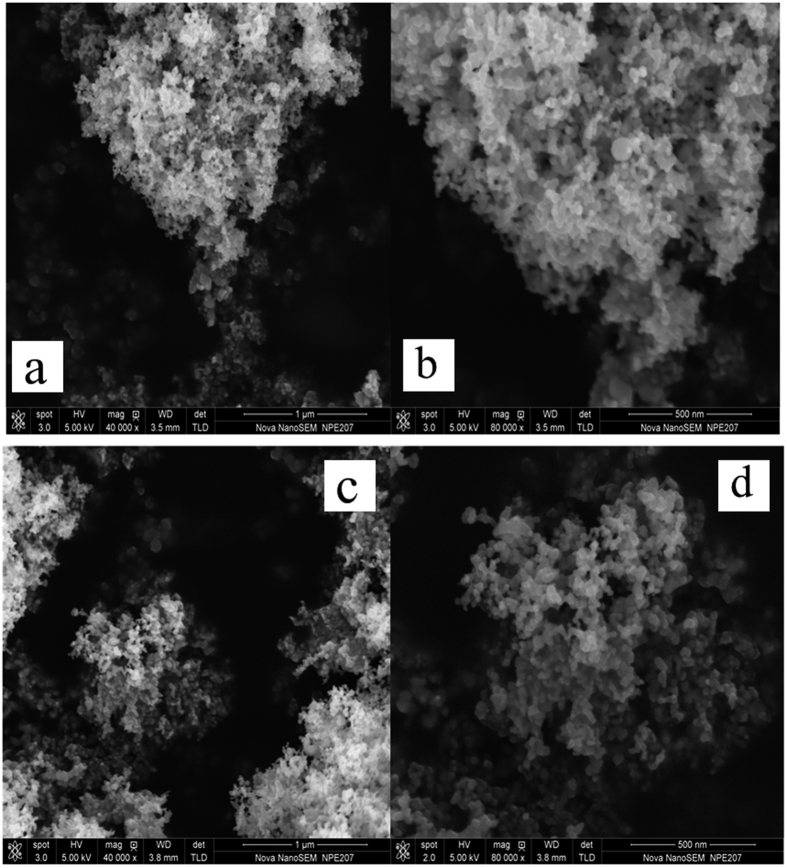
SEM images of (**a**,**b**) P25 TiO_2_ and (**c**,**d**) PCN4.

SEM images of P25 TiO_2_ and PCN4 in Fig. 4 suggest that the highly monodispersed particle distribution has been maintained after g-C_3_N_4_ self-assembly coating. PCN4 preserves a macroscopic network structure with a relatively regular array of macropores (Fig. 4d). The macropores also have similar sizes as pure TiO_2_. It is interesting that the surface of PCN4 is roughened after g-C_3_N_4_ coating. From the morphology comparison of bare P25 TiO_2_ and PCN4, similar highly-integrated composite systems can be observed, indicating a homologous surface area of PCN4 with P25, which is in good agreement with the BET results. In general, the particles of PCN4 are highly-monodispersed, which benefits its application in phototherapy.

**Figure 5:**
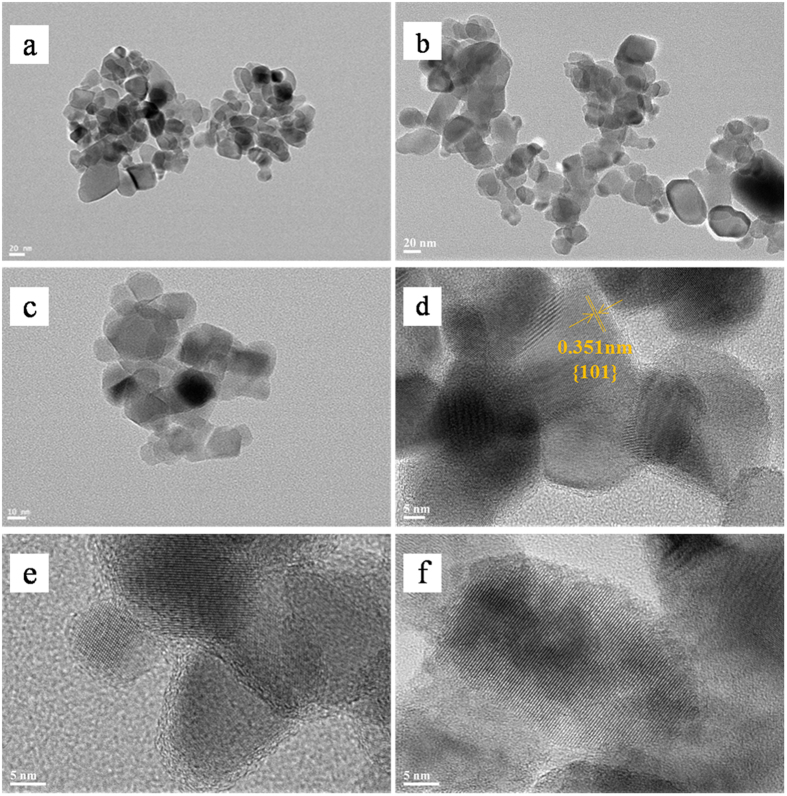
TEM images of (**a**) P25 TiO_2_ and (**b**) PCN4, and high-resolution TEM images of (**c**) P25, (**d**) PCN2, (**e**) PCN4 and (**f**) PCN8.

The morphological and structure of PCNx heterojunctions were further investigated by transmission electron microscopy (TEM) and high-resolution TEM (HRTEM). As showed in Fig. 5a,b, the particle sizes of TiO_2_ in pure P25 TiO_2_ and PCN4 both range from ca. 25 to 35 nm, which well agrees with the sizes of P25 TiO_2_ determined from XRD. The well-dispersed g-C_3_N_4_ layers are deposited all over the surface of P25 TiO_2_ nanoparticles. As showed on the high- resolution images of P25, PCN2, PCN4 and PCN8 (Fig. 5c–f), the lattice pitch of TiO_2_ in PCN4 is 0.351 nm, which is in accordance with (1 0 1) lattice plane character of anatase TiO_2_^24^ and similar to that of bare P25 TiO_2._ There is no difference on lattice pitch among the PCNx composites, indicating our synthetic method is moderate and does not affect the lattice stability of P25 TiO_2_. It should be noted that a close coating layer appears uniformly on the surface of TiO_2_ nanoparticles, which is probably formed by the stacking of g-C_3_N_4_ nanosheets (Fig. 5b). The g-C_3_N_4_ coating shells of PCN2, PCN4 and PCN8 are about 1, 2 and 3 nm thick, respectively. Hence, the layered shells are thickened with the increase of g-C_3_N_4_:TiO_2_ mass ratio. These g-C_3_N_4_ shells not only keep a biocompatible metal-free surface, but also form g-C_3_N_4_/TiO_2_ heterojunctions with excellent VLD photocatalytic performance. However, an excess of g-C_3_N_4_ coating shell will reduce the light harvest of g-C_3_N_4_/TiO_2_ heterojunctions and block the transfer channel of mass and free radical during photocatalysis, which will be discussed in detail later.

### FT-IR spectra

**Figure 6:**
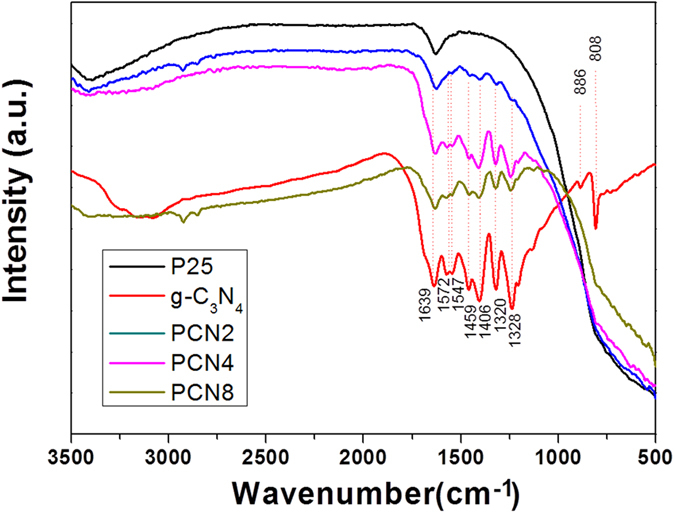
FT-IR spectra of P25 TiO_2_, pure g-C3N4, PCN2, PCN4 and PCN8.

Figure 6 shows the FT-IR spectra of pure g-C_3_N_4_, PCN2, PCN4 and PCN8. For pure P25 TiO_2_, the main peaks at 400–700 cm^−1^ are assigned to the stretching vibrations of Ti-O-Ti and Ti-O in anatase crystals, while the other two wide peaks at 1650 and 3400–3500 cm^−1^ correspond to hydroxyl group and physically-adsorbed water, respectively. The spectrum of pure g-C_3_N_4_ shows the strong bands within 1200–1650 cm^−1^, with peaks at 1238, 1320, 1406, 1459, 1547, 1572 and 1639 cm^−1^, which correspond to the typical stretching vibrations of the sp3 C-N bonds and sp2 C = N heterocycles^25^. Additionally, the peak at 807 cm^−1^ is due to the out-of-plane skeletal breathing of triazine. Besides, a wide band between 3000 and 3400 cm^−1^ corresponds to the N-H stretching vibration of residual NH_2_ attached to the sp2-hybridized carbon^26^. The NH_2_ band is not obvious in PCNx. Nevertheless, the stretching vibration intensification of C-N and C = N heterocycles in the spectra clearly indicates the presence of g-C_3_N_4_ with the increase of the g-C_3_N_4_/TiO_2_ ratio.

### Ultraviolet-visible diffuse reflectance spectroscopy (UV–vis DRS)

**Figure 7:**
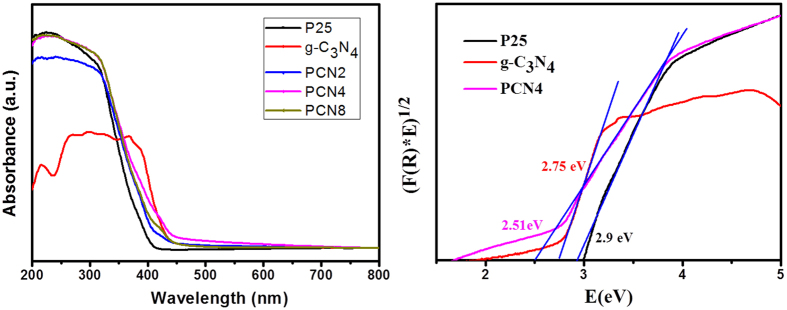
UV–vis diffuse reflectance spectra and theestimated band gap of the photocatalysts.

Figure 7 shows the UV–vis DRS spectra of PCNx. The strong absorption throughout the UV region is characteristic of TiO_2_. For bare P25 TiO_2_ and pristine g-C_3_N_4_, the estimated band gaps are 2.90 and 2.75 eV, respectively. It can be inferred from DRS that in addition to the typical absorption band of TiO_2_, a second small shoulder appears at higher wavelength in the spectra of PCNx. This means PCNx photocatalysts exhibit broader absorption and narrower band gap, owing to the formation of g-C_3_N_4_/TiO_2_ heterojunctions^27^. The absorption area of PCNx in the green light range (490–550 nm) is enlarged, which provides a basic possibility for catalyst-assisted phototherapy under green light irradiation. The band gap energies of indirect semiconductors can be estimated by Kubelka−Munk transformation. The calculated band gap values also change with the increase of g-C_3_N_4_ content (Table 1). PCN4 has the most optimized band gap of 2.51 eV. Clearly, the resulting g-C_3_N_4_/TiO_2_ functional material shows an enhanced capacity of green light harvest due to the narrow band gap of g-C_3_N_4_/TiO_2_ heterojunctions, especially for PCN4.

### Photocatalytic activity and photostability under green light irradiation

**Figure 8:**
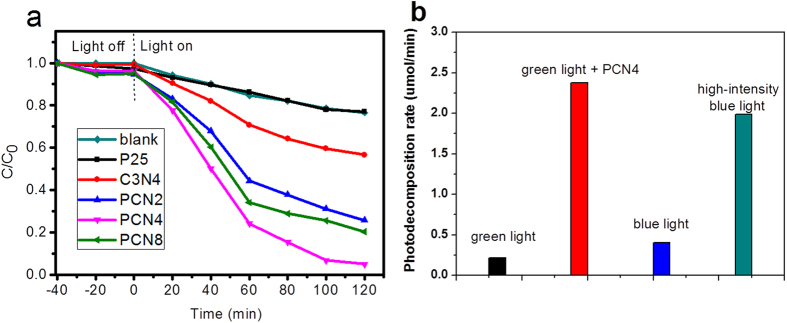
(**a**) Photodecomposition of 300 umol·L^−1^ bilirubin over P25 TiO_2_, pure g-C_3_N_4_, PCN2, PCN4 and PCN8 and (**b**) Comparative photocatalytic effect of PCN4 with other protocols all under 595 nm green light irradiation (5 mW·cm^2^).

The photocatalytic activities of the PCNx were evaluated by bilirubin degradation experiments using a 300 umol·L^−1^ bilirubin solution, in which the bilirubin concentration was similar to that in the blood of neonatal jaundice patients. The irradiation intensity was set at 5 mW/cm^2^. Figure 8a shows the bilirubin degradation curves in aqueous solutions with the presence of a photocatalyst under 495 nm green light irradiation, where *C*_*0*_ and *C* are the bilirubin concentrations under equilibrium and after visible-light irradiation, respectively. Prior to irradiation, the bilirubin solutions added with catalyst were kept in the dark for 40 min to reach adsorption equilibrium. Also a bilirubin solution without addition of any catalyst was set as a blank control. Results show the bilirubin degradation was very low in the dark or under green light irradiation without any photocatalyst, indicating the self-degradation of bilirubin was not obvious. Meanwhile, pure P25 and g-C_3_N_4_ also present rather low bilirubin degradation rates. However, the thin g-C_3_N_4_-modified TiO_2_ photocatalysts were much more photoresponsive to green light irradiation. Bilirubin photoalteration was significantly promoted with the presence of PCNx under green light, in which bilirubin concentration steeply decreased by ~70% after 120 min. The bilirubin degradation efficiencies after 60 min were 15.3%, 13.7%, 29.2%, 55.7%, 75.9% and 65.9% in the blank and with the presence of P25, g-C_3_N_4_, PCN2, PCN4 and PCN8, respectively, indicating that bilirubin was degraded more efficiently by PCNx than by the pure P25 or g-C_3_N_4_. Over the clinically relevant normal limit of 100 umol·L^−1^, the decrease of bilirubin concentration was drastic and significant, indicating the reaction follows a pseudo-first-order procedure. Among all the PCN catalysts, the optimum carbon nitride content envisaged was found in PCN4, which nearly photoreduced half of bilirubin only after 40 min. It is noticeable that the physical mixing of TiO_2_ and g-C_3_N_4_ led to a similar photoactivity of pure TiO_2_. This fact clearly denotes a synergetic effect to impregnation preparation, which provides a better junction between the two catalysts. Provided that the reaction follows a pseudo-first-order procedure, we estimated the bilirubin decomposition rate of PCN4 was 2.38 umol·min^−1^, much higher than the blank, P25 and g-C_3_N_4_ (0.59, 0.57 and 1.09 umol·min^−1^, respectively). Therefore, photocatalytic bilirubin degradation was successfully achieved by g-C_3_N_4_/TiO_2_ heterojunctions under illumination of 495 nm green light.

Regarding the safety requirement in biochemical applications, we further evaluated the stability of pure g-C_3_N_4_, bare P25 TiO_2_ and PCN4 through reuse experiments after bilirubin photodegradation (Figure S2). After five cycles of bilirubin photocatalytic degradation, PCN4 remained high activity, confirming that the g-C_3_N_4_/TiO_2_ heterojunctions were effective and stable during the photocatalysis. The comparison of XRD patterns between fresh PCN4 and used PCN4 (Figure S3) shows almost no difference, which demonstrates the excellent structural stability of the g-C_3_N_4_/TiO_2_ heterojunctions.

### Comparative photocatalytic activity under different irradiations

To compare the practical values in bilirubin degradation between photocatalyst-assisted green light phototherapy and the currently-used blue light-induced phototherapy, we recorded the photocatalytic performances of all systems using different light sources and photocatalysts (Fig. 8b). In addition, a 20 mW/cm^2^ blue light source was introduced to simulate the high-intensity blue light phototherapy condition. Clearly, the photodegradation rate under 5 mW/cm^2^ green light without any catalyst was rather low. The bilirubin photodegradation rate under 5 mW/cm^2^ blue light was two times of that under 5 mW/cm^2^ green light irradiation. However, the photodegradation effect under 5 mW green light irradiation was promoted significantly by PCN4. The bilirubin degradation activity with the presence of PCN4 under 5 mW green light was almost 6 times that under 5 mW/cm^2^ blue light irradiation without catalyst, and was even better than the effect under 20 mW/cm^2^ high-intensity blue lightirradiation without any photocatalyst. This result confirms that the PCNx can affect the efficacy of bilirubin phototherapy, which provides a new promising path based on moderate green light irradiation.

### Mechanisms of photocatalytic activity enhancement

Generally, photocatalytic activity is mainly governed by surface properties, light-absorption ability and photogenerated charge-separation efficiency. The BET experiments above indicate that the coating of g-C_3_N_4_ does not substantially affect the SSAs of PCNx compared with P25 TiO_2_. However, the light-absorption ability was improved by g-C_3_N_4_ coating treatment in forming g-C_3_N4/TiO_2_ heterojunctions, as indicated by UV–vis DRS. The photogenerated charge separation process was investigated by electrochemical impedance spectroscopy (EIS). The arc radius on EIS Nyquist plot of PCN4 under green light irradiation is smaller than that of g-C_3_N_4_ and P25 (Figure S5), indicating the effective separation of photogenerated electron-hole pairs and fast interfacial charge transfer in g-C_3_N_4_/TiO_2_ heterojunctions under green light excitation. Therefore, the close g-C_3_N_4_ and TiO_2_ interaction is thus an important influence factor on the photocatalytic activity of TiO_2_. Meanwhile, the amount of g-C_3_N_4_ in PCNx should be prudently controlled. When the g-C_3_N_4_ coating was very low as in PCN2, the photogenerated charges cannot efficiently migrate due to the poor electroconductivity of g-C_3_N_4_, thus reducing the heterojunction effect. However, with excessive g-C_3_N_4_ as in PCN8, a dense g-C_3_N_4_ shell around TiO_2_ was gradually formed, which prevented TiO_2_ from harvesting light energy and led to a serious recombination of electron-hole pairs. The optimum coating of g-C_3_N_4_ was 4 wt.%, which enhanced photocatalytic activity and inhibited photocorrosion. This mechanism of photocatalytic activity enhancement was approved by former reports^15^.

**Figure 9:**
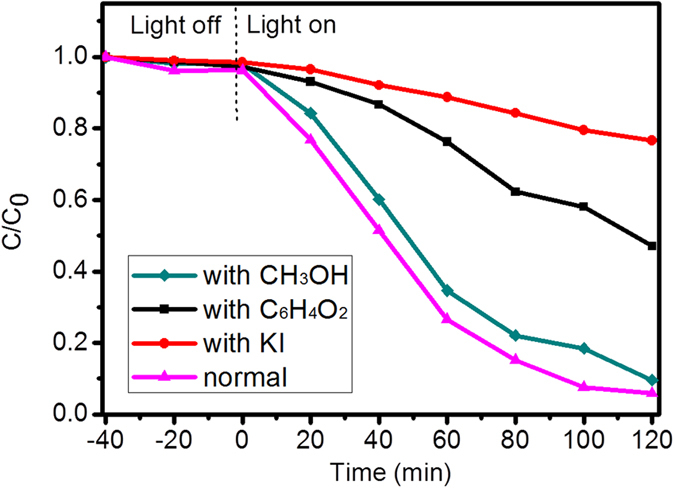
Plots of photogenerated carriers trapped during the photocatalytic degradation of bilirubin.

To investigate and identify the main active oxidation species generated in the photocatalytic process responsible for the degradation of bilirubin under green light, radical trapping experiments were performed in the presence of KI (a trapping scavenger for h^+^ and ⋅OH radicals on the catalyst surface), methanol (CH_3_OH, a trapping scavenger for ⋅OH radicals in the solution) and 1,4-benzoquinone (C_6_H_4_O_2_, a trapping scavenger for O⋅^2−^), respectively. The results shown in Fig. 9 indicate that the degradation efficiencies of bilirubin significantly decreased from 94.1% to 23.4% after 120 min in the presence of 4 mM KI. When 4 mM C_6_H_4_O_2_ was added to the reaction system, the degradation efficiency of bilirubin slightly decreased to 52.9%. However, 100 mM CH_3_OH just affect the degradation efficiency a little. These results indicated that h^+^ on the catalyst surface is the most important oxidising species during the photocatalytic process, that O⋅^2−^ radical is also responsible for the degradation of bilirubin, and that ⋅OH radical in the solution is not the main active species.

As far as we know, due to the narrow band gap of 2.67 eV and a relative more negative CB position of approximately −1.1 eV of g-C_3_N_4_, the photogenerated charges separation and transfer model in g-C_3_N_4_/TiO_2_ can follow heterojunction-type or Z-scheme mechanism^28^,^29^,^30^. Comparing these two models, the active species in the Z-scheme model are photogenerated hole, superoxide anion radical and hydroxyl radical, but the active species of heterojunction-type model only involves superoxide anion radical. In this case, the reactive species trapping experiment results clearly demonstrated that the holes play the leading role in photocatalytic remove of bilirubin, while no obvious ⋅OH radical can be found. The main reason was that the VB potential of CN is lower than that of the normal potential of the OH^−^/⋅OH ( + 2.4 V versus NHE), thus the photo-generated holes on the surface of g-C_3_N_4_ cannot react with OH^−^/H_2_O to form ⋅OH radical. Therefore, we propose the separation and transfer model of as-prepared Core/Shell P25/g-C_3_N_4_ under green light was a Z-scheme, in which photogenerated holes directly consumed by the bilirubin molecules attract on the surface of g-C_3_N_4_/TiO_2_. This is in accordance with previous reports^31^,^32^.

### Prospects of clinical application

**Figure 10:**
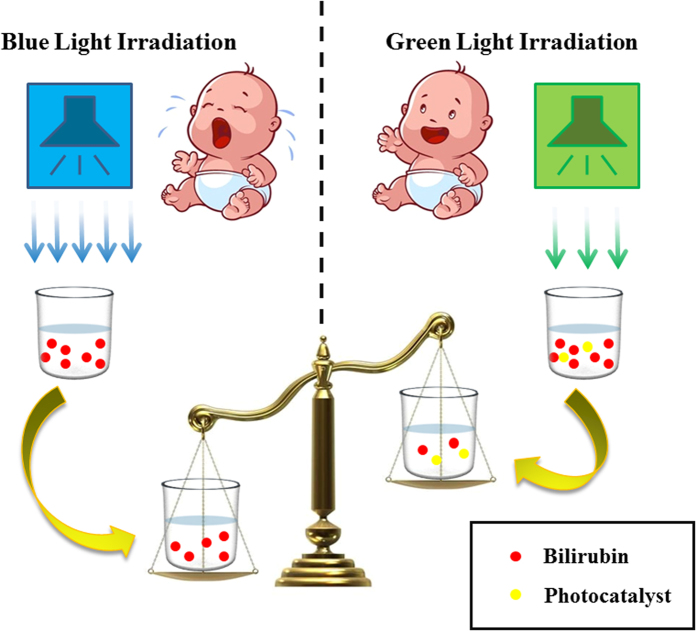
Illustration of the advantage and prospect of photocatalyst-assisted jaundice therapeutic protocols.

In this work, the more important issue was to clarify the mechanism and potential of the therapeutic protocols of photocatalyst-assisted bilirubin removal under moderate green light irradiation compared with the high-intensity blue-light-driven phototherapy. Previous animal experiments showed that skin bilirubin of jaundiced rats under blue or green light irradiation was converted to metastable geometric isomers, which were then transported in the blood and excreted in the bile^33^,^34^. The same reaction probably occurred in light-exposed jaundiced babies, particularly during phototherapy. Since blue light irradiation can also be strongly absorbed by competing hemoglobin which is rich in human blood, the optical intensity in blue light therapy must be high enough to ensure the bilirubin molecules receive enough energy for efficient photoalteration^35^. The undesirable high-intensity irradiation causes health problems such as hyperthermia, insensible water loss, rash and even DNA damages. The use of bio-tolerant green light is attractive regarding the health protection of newborn babies which is the most popular in neonatal jaundice patients (Fig. 10). In this work, photocatalysis experiments show g-C_3_N_4_/TiO_2_ heterojunction can improve the efficacy of bilirubin phototherapy under relatively moderate green light irradiation. Our preliminary toxicology experiments in mouse fibroblast cell line L-929 showed that the toxicity of PCNx was between grade 0 and 1, and the material lacked hemolytic activity, which indicates the high biocompatibility of this g-C_3_N4/TiO_2_ heterojunction. Therefore, the g-C_3_N4/TiO_2_ hybrids here are suitable for further biochemical applications. This provides a new pathway to design photocatalyst-assisted neonatal jaundice therapeutic protocols based on moderate green light as an ideal irradiation source in the future, such as services as photosensitizer in photodynamic therapy, undissolved injectable *in-situ* forming implants or non-invasive bleaching membrane^36^,^37^.

## Conclusions

The g-C_3_N_4_/TiO_2_ heterojunctions with metal-free surfaces were synthesized through a simple impregnation route. Multiple characterization techniques revealed that the close integration of g-C_3_N_4_ and TiO_2_ in g-C_3_N_4_/TiO_2_ heterojunction extended its visible light response and promoted its charge-separation efficiency. The g-C_3_N_4_/TiO_2_ nanocomposites were used as high-performance photocatalysts for neonatal jaundice phototherapy by photocatalytic removal of bilirubin under green light irradiation. The 4 wt.% g-C_3_N_4_/TiO_2_ heterojunction demonstrated a remarkable photocatalytic performance of 50% bilirubin removal rate under 495 nm green light within 40 min, as well as an excellent cycle performance. The enhancement of photocatalytic activity after coating may be mainly attributed to the Z-scheme synergic effect between g-C_3_N_4_ and TiO_2_, which enhances the green light harvest and greatly accelerates the separation of photogenerated carriers. The bilirubin removal effect with the presence of g-C_3_N_4_/TiO_2_ photocatalysts was clearly higher than that under clinically-used high- intensity blue light without any photocatalyst. These findings provide insight of an instant, noninvasive and cheap neonatal jaundice treatment protocol of semiconductor photocatalyst-assisted phototherapy under moderate green light irradiation.

## Methods

### Materials

P25 TiO_2_ (Degussa Co., LTD., Germany) was used as the substrate for g-C_3_N_4_ self-assembly coating. Bilirubin (Sigma-Aldrich Co., LTD, USA), melamine and methanol (analytical grade, Shanghai Chemical Corp.) were used without further purification.

### Preparation of g-C_3_N_4_/TiO_2_ photocatalysts

The g-C_3_N_4_/TiO_2_ heterojunctions were prepared according to a reported procedure except using P25 TiO2 as the substrate. Firstly, bulk g-C_3_N_4_ was prepared by simple calcination of melamine at 550 °C for 4 h in a covered alumina crucible. Then 0.1 g of g-C_3_N_4_ was added into 100 mL of methanol and sonicated for 2 h to make the bulk g-C_3_N_4_ exfoliated into g-C_3_N_4_ thin sheets, forming suspension A. Meanwhile, 1 g of P25 TiO_2_ power was added into 100 mL of methanol and sonicated for 10 min, forming suspension B. The g-C_3_N_4_/TiO_2_ heterojunctions were synthesized by simply mixing appropriate amounts suspension A and suspension B together and stirring them at room temperature in the dark for 24 h. Afterwards, the mixtures were evaporated at room temperature to remove the methanol, forming composite photocatalysts.

### Characterization

XRD patterns were recorded on a Rigaku D/MAX-2495VB/PC diffractometer under the following conditions: θ–2θ mode, CuKα_1_ radiation (λ = 1.5406 Å), 40 kV, 100 mA, and scanning step 0.02 ° per sec. The average crystal size was determined from XRD pattern parameters according to the Scherrer equation. SEM images were recorded using an FEI XL-30 scanning electron microscope, operated at an acceleration voltage of 25 kV. TEM images were taken on a JEOL JEM-2010 TEM device with an acceleration voltage of 200 kV. The Brumauer–Emmett–Teller (BET) specific surface areas were calculated based on nitrogen sorption isotherms which were recorded on a BeiShiDe 3H-2000PS4 device at −196 °C, pore size distributions were deduced via the Barrett– Joyner–Halenda (BJH) method. Total pore volume (V_t_) was determined at a relative pressure of 0.98. XPS data were recorded by a PHI 5000 C ESCA X-ray photoelectron spectrometer with Al Kαsource at 14.0 kV and 25 mA. All the binding energies were referenced to the contaminant C 1 s peak at 284.6 eV. The ultraviolent–visible (UV–vis) diffuse reflectance spectra (DRS) were achieved using a Shimadzu UV-2401 UV–vis spectrometer equipped with integrating sphere. Fourier transform infrared (FTIR) spectra were tested on a Nicolet Nexus 470 FTIR Spectrometer. Electro-chemical impedance spectroscopy (EIS) tests were performed at open circuit potential over the frequency range between 100 kHz and 0.1 Hz.

### Evaluation of photocatalytic activities

The photocatalytic performances of g-C_3_N_4_/TiO_2_ photocatalysts were determined by degrading bilirubin under 595 nm monochromatic green lights from a 100 W lamp which was powered by an LED system (CEL-LED100, Beijing CEAULIGHT Co. Ltd.). The excitation intensity can be controlled by tuning the applied current of the lamp and determined by a LED luminous intensity meter (ALTT-86LB, Beijing CEAULIGHT Co. Ltd.). Photocatalytic degradation of bilirubin was performed in a bilirubin solution (50 mL) with an initial concentration of 300 umol·L^−1^. The bilirubin solution was stirred in the dark after the photocatalysts were added for 40 min until adsorption-desorption were balanced between the catalyst and contaminants. Then the resulting mixtures were exposed to green light. Afterwards, 1 mL of aliquots were periodically withdrawn from the reaction vessel every 20 min, diluted and centrifuged at 10,000 rpm for 5 min to separate the catalysts. Bilirubin levels in solution were measured by a UV–vis spectrophotometer (Spectrumlab 752 s, Xunda, Shanghai) at λ = 483 nm. The photocatalytic decolorization is a pseudo- first-order reaction: ln(*C*_0_/*C*_t_) = *k*t, where the *k* is the apparent rate constant, *C*_0_ and *C*_t_ represent the concentration of bilirubin at initial stage and after irradiation for some time, respectively. For comparison, the bilirubin removal rate under blue light was investigated by using a monochromatic LED light of 440 nm from the 100 W LED lamp by the same method. In the photocatalytic stability evaluation, the reaction photocatalysts were washed by centrifugation at the end of each cycle to remove the organic residues.

## Additional Information

**How to cite this article**: Kang, S. *et al*. Efficient Photocatalytic Bilirubin Removal over the Biocompatible Core/Shell P25/g-C_3_N_4_ Heterojunctions with Metal-free Exposed Surfaces under Moderate Green Light Irradiation. *Sci. Rep.* **7**, 44338; doi: 10.1038/srep44338 (2017).

**Publisher's note:** Springer Nature remains neutral with regard to jurisdictional claims in published maps and institutional affiliations.

## Supplementary Material

Supplementary Information

## Figures and Tables

**Table 1:** Textural properties and energy band gap (Eg) of g-C_3_N_4_ and PCNx catalysts.

Sample	Mass ratio of g-C_3_N_4_ and TiO_2_ (%)	S_BET_ (m^2^/g)	Pore size (nm)	Pore volume (cm^3^/g)	Eg (eV)
P25 TiO_2_	0	54.28	19.74	0.3165	2.90
PCN2	2	42.18	6.18	0.0651	2.57
PCN4	4	45.79	6.40	0.0731	2.51
PCN8	8	44.83	6.34	0.0710	2.59
g-C_3_N_4_	—	20.04	98.91	0.4955	2.75
