# Supplementary material for: Efficient Photocatalytic Bilirubin Removal over the Biocompatible Core/Shell P25/g-C3N4 Heterojunctions with Metal-free Exposed Surfaces under Moderate Green Light Irradiation
Source: Sci Rep. 2017 Mar 13;7:44338. doi: 10.1038/srep44338 (PMC5347124; doi:10.1038/srep44338)
Supplement: Supplementary Information [file srep44338-s1.doc]

Supplementary Information for

Efficient Photocatalytic Bilirubin Removal over the Biocompatible Core/Shell P25/g-C3N4 Heterojunctions with Metal-free Exposed Surfaces under Moderate Green Light Irradiation

**Shifei Kang^1^**†**, Hengfei Qin^3^**†**, Yongkui Huang^4^, Lu Zhang^1^, Xia Bai^1^, Xi Li^4^, Di Sun^2^*,Yangang Wang^1^*,Lifeng Cui^1^***

**^1^**Department of Environmental Science and Engineering, University of Shanghai for Science and Technology, Shanghai, 200093, China.

**^2^**Department of Ultrasound in Medicine, Shanghai Jiao tong University Affiliated Sixth People's Hospital, Shanghai Institute of Ultrasound in Medicine, Shanghai 200233, China

**^3^**School of Chemical and Environmental Engineering, Jiangsu University of Technology, Changzhou, 213001, China.

**^4^**Department of Environmental Science and Engineering, Fudan University, Shanghai 200433, China.

† *S.F.K. and Q.H.F contributed equally.*

** Correspondence andrequests for materials should be addressed to D.S.* *(email:sundy316@163.com), Y.G.W (email:ygwang8136@gmail.com) or L.F.C (email:*[*lifeng.cui@gmail.com*](mailto:lifeng.cui@gmail.com)*).*

1. XPS spectra of PCN4

Figure S1 XPS spectra of representative PCN4 sample: (a) survey scan spectrum and high-resolution spectra of (b) Ti 2p, (c) O 1s, (d) C 1s, and (e) N 1s.

2. Photocatalytic stability evaluation of PCN4

Figure S2 Photocatalytic time course of PCN4 sample for bilirubin photodecomposition in solutions (300 umol·L^−1^) under 595 nm green light irradiation (5 mW·cm^2^).

3. XRD patterns of fresh PCN4 and used PCN4.

Figure S3 Comparison on XRD patterns of fresh PCN4 and used PCN4 for photodecomposition against bilirubin after 5 cycles.

4. Electro-chemical impedance spectroscopy (EIS) tests results

Figure S4 EIS Curves of P25 TiO_2_, pure g-C_3_N_4_ and PCN4.
